# To the Editors of the Medical and Physical Journal

**Published:** 1800-02

**Authors:** W. Humby

**Affiliations:** St Alban's Street


### *To the Editors of the Medical and Phyfical Journal.*

Gentlemen,

THE following Cafe of *Lusus Naturæ*, may, probably, be worthy a place in your valuable Journal, and not unacceptable to its numerous Readers.

In October laft, I was engaged to attend the wife of a refpectable tradefman, who expected to be confined about the end of November, or beginning of December. I heard nothing of her till two in the afternoon of the 12th inftant, when I was informed fhe was unwell, and expected foon to need my affiftance. At half paft three I was defired to vifit her; I went directly; and on hearing her voice, was convinced that the pain fhe was then fuffering was an expelling one. I found the head and fhoulders born; they were involved in the membranes, which I ruptured at the neck, and flipt over the head; the child at this time apparently lifelefs. I determined to wait the natural expulfion of the breech, &c. rather than haften the delivery. In about five minutes, I received a mafs of a very uncommon nature; the placenta I diftinctly felt; I withdrew the whole with the child, and wrapt it in a flannel which lay at hand. My attention was then directed to the mother, fearful of hæmorrhage from fo fudden a feparation of the placenta. I proceeded to afcertain the ftate of the uterus, and difcovered that its fundus was to be felt by deep preffure only, on a line with the pubes. I was now fatisfied with refpect to the fafety of my patient, and proceeded to infpect the child; its appearance was fo extraordinary that I was defirous of taking a more minute examination than I poffibly could in the chamber. I was permitted to take it away in the evening; and on examination, in the prefence of Dr. Ainflie, Mr. Earl, and Mr. Heavifide, the appearances were as follow:

The head, perfect and natural; the thorax, very fmall, with a depreffion of the coftæ on the right fide; the arms perfect, but a ftricture in the elbow joints, which prevented their full extenfion, (this ftricture attracted my notice immediately on rupturing the membranes;) the fpine fhorter than ufual, curved to the left fide, fo that the facrum, &c. were in a horizontal pofition; the lower limbs, diftorted and contracted; the left foot, perfect; the right, fhapelefs, with two toes only; but the moft remarkable appearance was, the total want of abdominal mufcles and integuments; the liver, ftomach, and inteftines, (the larger ones diftended with meconium) expofed; no omentum; the peritonæum, emanating from the thorax and curvature of the fpine, hung over them as an apron, having no attachment at its inferior edge; the funis not more than fix inches long; the placenta, natural, excepting a particular fatty appearance at its circular edge.*

The mother of this extraordinary production (which was of the male fpecies,) does not recollect having met with any accident in the early part of pregnancy; fhe was fuckling at the time fhe became pregnant, which accounts for the error in her reckoning. She quickened about the ufual time, and was fenfible of the motion of the child, till within two days of delivery. She has had feveral children, but her feelings in this cafe fhe defcribes as very different from any fhe before experienced.

I am, Gentlemen,

Your's, refpectfully,

W. HUMBY.

*St Alban's Street, Jan. 15th,* 1800.

---

### *To the Editors of the Medical and Phyfical Journal.*

* The preparation of this uncommon fubject may be feen in Mr. Heavifide's elaborate collection.

